# Der Zusammenhang zwischen Thiazolidinedionen (Glitazonen) und Blasenkrebs: Ein Rapid Review

**DOI:** 10.1007/s00120-025-02557-x

**Published:** 2025-03-11

**Authors:** Laila Schneidewind, Barbara Sommerhalder, Dario Willi, Cindy Rönnau, Annemarie Uhlig, Bernhard Kiss

**Affiliations:** 1 Universitätsklinik für Urologie, Universität Bern, Inselspital Bern, Bern, Schweiz; 2 Klinik und Poliklinik für Urologie, Universitätsmedizin Greifswald, Greifswald, Deutschland; 3 Klinik für Urologie, Universitätsmedizin Göttingen, Göttingen, Deutschland; 4 Universitätsklinik für Urologie, Universität Bern, Inselspital, Wilhelm-Fabry-Haus, Freiburgstr. 37, 3010 Bern, Schweiz

**Keywords:** Harnblasenkarzinom, Diabetes mellitus, Glitazone, Rosiglitazon, PPAR‑γ Agonisten, Bladder cancer, Diabetes mellitus, Glitazone, Rosiglitazone, PPAR‑γ agonists

## Abstract

**Hintergrund:**

Seit der Zulassung von Glitazonen als orale Antidiabetika wird kontrovers diskutiert, ob diese das Risiko für Blasenkrebs erhöhen oder dessen Progression verstärken. Aktuelle Daten zeigen, dass Glitazone die Expression von Nectin4 hochregulieren können.

**Fragestellung:**

Diese Ergebnisse machen eine erneute detaillierte Auseinandersetzung mit der Thematik unumgänglich. Konsequenterweise führten wir daher eine schnelle Evidenzanalyse durch. Primär sollten systematische Übersichtsarbeiten gezeigt werden, die diesen Zusammenhang untersuchen. Sekundär sollten dann translationale Studien identifiziert werden, die den molekularbiologischen Hintergrund beschreiben.

**Material und Methoden:**

Es wurde eine schnelle Evidenzanalyse mit Literaturrecherche in MEDLINE via PubMed für den Zeitraum Juli 2000 (Erstzulassung von Glitazonen EU) bis zum Datum der letzten Suche (15. Juli 2024) durchgeführt.

**Ergebnisse:**

Die primäre Literatursuche ergab 860 Treffer, schließlich konnten 14 Studien eingeschlossen werden. Davon sind 6 systematische Übersichtsarbeiten (5 inklusive Metaanalyse) und 8 translationale Studien. In den Übersichtarbeiten bleibt die Datenlage bzgl. der Assoziation von Glitazonen mit Blasenkrebs uneindeutig, insbesondere durch die Heterogenität der zugrunde liegenden Studien sowie zahlreiche Störfaktoren wie Alter oder Raucheranamnese. Ein experimenteller Nachweis eines kausalen Zusammenhangs von Glitazonen und Blasenkrebs konnte nicht erbracht werden. Zwei inkludierte Studien sehen sogar einen möglichen therapeutischen Nutzen der Präparate bei diesen Patienten.

**Schlussfolgerung:**

Es bleibt weiterhin unklar, ob Glitazone in der Entstehung von Harnblasenkarzinomen eine relevante Rolle spielen. Der mögliche therapeutische Nutzen sollte dringend weiter untersucht werden.

## Abstract (en)

**Background:**

Since the authorization of glitazones as oral antidiabetics, there has been a controversial debate as to whether they increase the risk of bladder cancer or accelerate its progression. Recent data have shown that glitazones can upregulate the expression of Nectin4.

**Objectives:**

These results make a renewed detailed examination of the topic unavoidable. Consequently, we conducted a rapid evidence analysis. The primarily aim was to identify systematic reviews investigating this relationship. Second, translational studies that describe the molecular biological background were identified.

**Materials and methods:**

A rapid evidence analysis was conducted with a literature search in MEDLINE via PubMed for the period July 2000 (first approval of glitazones in the European Union) to the date of the last search (15 July 2024).

**Results:**

The primary literature search yielded 860 hits and ultimately 14 studies were included. Of these, 6 were systematic reviews (5 including meta-analyses) and 8 were translational studies. In the reviews, the data situation regarding the association of glitazones with bladder cancer remains ambiguous, particularly due to the heterogeneity of the underlying studies and numerous confounding factors such as age or smoking history. Experimental evidence of a causal relationship between glitazones and bladder cancer could not be demonstrated. Two included studies even see a possible therapeutic benefit of the preparations in these patients.

**Conclusion:**

It remains unclear whether glitazones play a relevant role in the development of bladder cancer. The possible therapeutic benefit should be urgently investigated further.

## Hintergrund und Fragestellung

Seit der Zulassung von Thiazolidindionen bzw. Glitazonen (die PPAR-γ-agonistisch wirksam sind) als orale Antidiabetika vor ca. 20 Jahren wird kontrovers diskutiert, ob diese das Risiko für Blasenkrebs erhöhen oder dessen Progression verstärken [1–4]. Im Juli 2010 gab das Bundesinstitut für Arzneimittel und Medizinprodukte (BfArM) eine Anwendungsbeschränkung für Rosiglitazon aufgrund des kardiovaskulären Risikoprofils heraus [5]. Ein Jahr später im Juli 2011 kamen vom BfArM weitere Kontraindikationen und Warnhinweise für Pioglitazon aufgrund des leicht erhöhten Blasenkrebsrisikos hinzu. Dort heißt es, dass Risikofaktoren für Blasenkrebs wie Alter, Raucherstatus oder Kontakt zu bestimmten Chemikalien, z. B. aromatischen Aminen, bei der Entscheidung für die Behandlung mit Pioglitazon berücksichtigt werden sollten. Pioglitazon sollte nur dann angewendet werden, wenn die Patienten eindeutig von der Behandlung profitieren. Dies ist, 3 bis 6 Monate nach Therapiebeginn und dann in regelmäßigen Abständen zu kontrollieren. Die Entscheidung für eine Pioglitazon-Therapie sollte unter Berücksichtigung der altersbedingten Risikofaktoren bei älteren Patienten besonders vorsichtig getroffen werden. Bereits hier wird zusätzlich vom BfArM gefordert, dass der Zulassungsinhaber eine europäische Studie zur Klärung der offenen Fragen bzgl. des Zusammenhangs mit Blasenkrebs durchführen sollte [6]. Dies blieb bisher aus und Pioglitazon ist zum aktuellen Zeitpunkt das einzig verfügbare Glitazon in der oralen Therapie des Diabetes mellitus in westlichen Industrieländern [1].

Allerdings bleibt der Sachverhalt dieses Zusammenhangs bis zum aktuellen Zeitpunkt weiterhin unklar und auch ein stringenter Kausalitätsbeweis zwischen der PPAR-γ-Aktivierung und der Induktion bzw. Progression von Blasenkrebs gelang bisher nicht. Im Gegenteil, es gibt sogar Berichte, dass PPAR-γ- Agonisten einen antiproliferativen Effekt auf Blasentumorzellen haben und somit sogar therapeutisch genutzt werden könnten [7]. Außerdem wurde auf dem Blasenkrebs-Kongress der American Association of Cancer Research (AACR) 2024 berichtet, dass Glitazone die Nectin4-Expression hochregulieren können und somit das therapeutische Fenster der Nectin4-basierten Therapien vergrößern könnten. Die Autoren eines dort vorgestellten Abstracts fassten zusammen: Die Modulation des PPAR-γ-Signalwegs erhöht die Expression von Nectin4, was wir mit Rosiglitazon genutzt haben, um die Antitumorwirksamkeit von Nectin4-CAR-T-Zellen zu erhöhen. Diese präklinischen Ergebnisse legen den Grundstein für die Entwicklung von CAR-T-Therapien bei Blasenkrebs und schlagen rationale Arzneimittelkombinationen vor, die das therapeutische Fenster von Therapien, die auf Nectin4 abzielen, erweitern können [8].

Diese Ergebnisse machen eine erneute detaillierte Auseinandersetzung mit der Thematik unumgänglich, um die Ergebnisse in der Therapie des Blasenkrebses weiter zu verbessern.

Konsequenterweise führten wir daher eine schnelle Evidenzanalyse zur Assoziation von Glitazonen mit Blasenkrebs durch. Primär sollten systematische Übersichtsarbeiten und Metaanalysen gezeigt werden, die diesen Zusammenhang untersuchen. Sekundär sollten dann experimentelle bzw. translationale Studien identifiziert werden, die eine mögliche Kausalität bzw. den molekularbiologischen Hintergrund beschreiben.

## Methodik

Es wurde eine schnelle Evidenzanalyse mit Literaturrecherche in MEDLINE via PubMed für den Zeitraum Juli 2000 (Erstzulassung von Glitazonen in der Europäischen Union) bis zum Datum der letzten Suche (15. Juli 2024) durchgeführt [9]. Als Suchbegriffe wurden die Begriffe „bladder cancer“, „diabetes mellitus“, „glitazone“, „PPAR‑γ agonist“ und „Nectin4“ sowie deren Kombination verwendet. Für die Evidenzsynthese wurden lediglich systematische Übersichtsarbeiten mit und ohne Metaanalyse sowie translationale Studien berücksichtigt. Bezüglich der Sprache wurden nur englische und deutsche Arbeiten eingeschlossen. Der primäre Endpunkt dieser Arbeit ist die Deskription der Assoziation von Glitazon-Derivaten mit dem Auftreten des Blasenkrebses. Sekundärer Endpunkt sollte die Darstellung molekularer Grundlagen, die eine mögliche Assoziation erklären könnten sein. Es wurden die PRISMA-Leitlinien zur Berichterstattung systematischer Übersichtsarbeiten angewandt [10].

## Ergebnisse

**Abb. 1:**
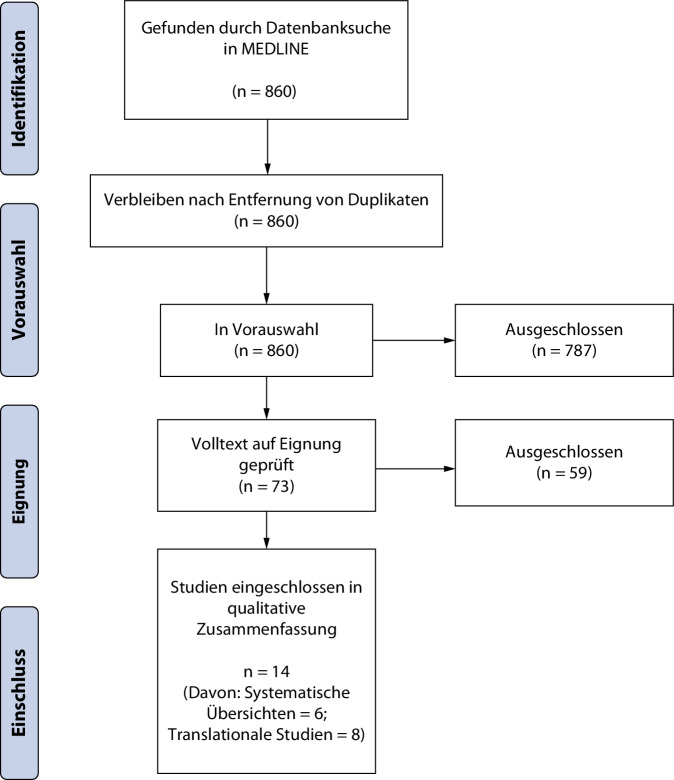
PRISMA-Flussdiagramm

Die primäre Literatursuche ergab 860 Treffer, schließlich konnten 14 Studien eingeschlossen werden. Dabei handelt es sich um 6 systematische Übersichtsarbeiten, 5 davon inklusive einer Metaanalyse und 8 translationale Untersuchungen. In der Abb. 1 findet sich das PRISMA-Flussdiagramm.

### Ergebnisse der systematischen Übersichtsarbeiten

Die Tab. 1 gibt einen Überblick über die Charakteristika der hier eingeschlossenen Arbeiten [1, 2, 11–14]. Die aktuellste Arbeit publiziert von Ripamonti et al. hat keine Metaanalyse vorgenommen und kommt zu der Schlussfolgerung, dass es angesichts der vorliegenden Daten nicht sinnvoll ist, die Ergebnisse sehr heterogener Studien zusammenzufassen. Konsequent durchgeführte Beobachtungsstudien sind erforderlich, um die Rolle der Anwendung von Pioglitazon auf das Auftreten von Blasenkrebs korrekt einzuschätzen zu können [1].

**Tab. 1:** Überblick und Charakterisierung der eingeschlossenen Übersichtsarbeiten (*n* = 6)

Referenz	Studiendesign	Hauptergebnisse	Schlussfolgerung der Autoren
*Systematisches Review (n* *=* *1)*			
Ripamonti et al., 2019 [1]	Systematische Übersichtsarbeit der Datenbanken MEDLINE und EMBASE im Zeitraum 1. Januar 2000 bis 30. Oktober 2017, prospektive und retrospektive Beobachtungsstudien eingeschlossen	Viele der Studien sind von verschiedenen Arten von Verzerrungen betroffen, insbesondere von zeitlichen Verzerrungen, die eine gepoolte Analyse ausschließen sollten, da dies zu einer verfälschten Schätzung des Risikos führen würde	Angesichts der vorliegenden Daten ist es nicht sinnvoll, die Ergebnisse sehr heterogener Studien zusammenzufassen, Rigoros durchgeführte Beobachtungsstudien sind erforderlich, um die Rolle der Anwendung von Pioglitazon auf das Auftreten von Blasenkrebs korrekt einzuschätzen
*Systematische Reviews/Metaanalyse (n* *=* *5)*			
Zhu et al., 2012 [11]	MEDLINE und EMBASE Suche, um Studien zu finden, die über die Wirkung von Pioglitazon auf Blasenkrebs bei Diabetikern berichteten; Zusammenfassende Effektschätzungen wurden mit Hilfe eines Metaanalysemodells mit festen Effekten abgeleitet	An 5 Studien nahmen 2.350.908 Diabetiker teil; Pioglitazon war verbunden mit einem Risiko für Blasenkrebs verbunden (relatives RR 1,17; 95 %-KI 1,03–1,32; *p* = 0,013); Noch stärker war der Effekt bei einer kumulativen Behandlungsdauer von > 24 Monaten (RR 1,38; 95 %-KI 1,12–1,70; *p* = 0,003). Es bestand ein signifikantes Risiko für Patienten mit einer kumulativen Dosis > 28.000 mg (RR 1,58; 95 %-KI 1,12–2,06; *p* = 0,001)	Die Behandlung mit Pioglitazon scheint mit einem deutlich erhöhten, Risiko für Blasenkrebs bei Patienten mit Diabetes mellitus einherzugehen
Ferwana et al., 2013 [12]	Elektronische Datenbanken (MEDLINE, EMBASE, CENTRAL) wurden abgefragt, um kontrollierte Studien zu Pioglitazon zu finden, in denen das Risiko von Harnblasenkrebs beschrieben wird	Sechs Studien mit 215.142 Patienten, die Pioglitazon einnahmen, wurden eingeschlossen, mit einer mittleren Nachbeobachtungszeit von 44 Monaten; das Risiko, an Blasenkrebs zu erkranken, war bei Patienten, die Pioglitazon einnahmen, signifikant höher (HR 1,23; 95%-KI 1,09–1,39; I^2^ = 0 %) im Vergleich zu den Kontrollgruppen	Patienten, die mit Pioglitazon behandelt werden, haben im Vergleich zur Allgemeinbevölkerung ein leicht erhöhtes Risiko für Blasenkrebs; bei Patienten mit Typ-2-Diabetes und Risikofaktoren wie familiärer Vorbelastung, Rauchen oder bestimmten Formen der Chemotherapie, müssen möglicherweise andere Antihyperglykämika in Betracht gezogen werden
Levin et al., 2015 [13]	Verordnungs‑, Krebs- und Sterblichkeitsdaten von Menschen mit Typ-2-Diabetes wurden aus sechs Populationen aus der ganzen Welt (British Columbia, Finnland, Manchester, Rotterdam, Schottland und dem UK Clinical Practice Research Datalink); diese wurden dann unter Verwendung fester und Metaregression mit zufälligen Effekten gepoolt analysiert	Es wurden Daten von 1,01 Mio. Personen über 5,9 Mio. Personenjahre eingeschlossen; es gab keine Hinweise auf einen Zusammenhang zwischen kumulativer Exposition gegenüber Pioglitazon und Blasenkrebs bei Männern (RR 100 Tage kumulativer Exposition 1,01; 95 %-KI 0,97–1,06) oder Frauen (RR 1,04; 95 %-KI 0,97–1,11) nach Anpassung für Alter, Kalenderjahr, Diabetesdauer, Rauchen und jeglicher Verwendung von Pioglitazon; es wurde kein Zusammenhang beobachtet zwischen Rosiglitazon und Blasenkrebs bei Männern (RR 1,01; 95 %-KI 0,98–1,03) oder Frauen (RR 1,00; 95 %-KI 0,94–1,07)	Die kumulative Anwendung von Pioglitazon oder Rosiglitazon war nicht mit dem Auftreten von Blasenkrebs in dieser großen, gepoolten Multipopulationsanalyse assoziiert
Qu et al., 2017 [14]	Embase, PubMed, Web of Science, Cochrane Central Register of Controlled Trials (CENTRAL) und ClinicalTrials.gov wurden von Anfang an bis zum 5. Januar 2017 ohne Spracheinschränkung durchsucht; Beobachtungsstudien und randomisierte kontrollierte Studien (RCT), die entweder relative Risikoschätzungen wie Risikoverhältnisse (RR), Hazard Ratios (HR) oder Odds Ratios (OR) und 95 %-Konfidenzintervalle (KI) für Krebserkrankungen oder Rohdaten lieferten, eingeschlossen	Insgesamt wurden 2.764.731 Teilnehmer aus Beobachtungsstudien (OB) und 9999 Teilnehmer aus RCT für diese Analysen identifiziert; die stratifizierte Analyse ergab, dass der Studientyp, die Anpassung für Alter/Geschlecht, Behandlungsdauer, kumulative Dosis, in einer Kontrollgruppe verwendete Wirkstoffe, durchschnittliche Nachbeobachtungszeit und Region der Studienpopulation zu den nicht übereinstimmenden Ergebnissen beitragen könnten	In Bezug auf die Bevölkerungsregionen, Pioglitazon das Risiko für Blasenkrebs erhöht, konnte in der europäischen Bevölkerung gefunden werden, und Patienten, die länger mit Pioglitazon behandelt werden (> 12 Monate) oder die länger mit Pioglitazon behandelt werden (> 12 Monate) oder denen eine höhere kumulative Dosis (> 28.000 mg) verabreicht wurde, benötigen möglicherweise mehr Aufmerksamkeit bzw. Sollten sorgfältiger überwacht werden
Mehtälä et al., 2019 [2]	Diese Metaanalyse basierte auf einer systematischen Überprüfung von begutachteten Beobachtungsstudien, die vor dem 30. September 2016 veröffentlicht wurden, Datenbank: MEDLINE	23 Studien wurden in diese Arbeit und 18 in die eigentlichen Metaanalysen einbezogen; bei Blasenkrebs betrug die geschätzte Effektgröße für die regelmäßige Einnahme von Pioglitazon im Vergleich zur Nicht-Einnahme von Pioglitazon 1,16 [95 %-KI 1,04–1,28]; das Risiko wurde jedoch im Bayes’schen Rahmen nicht verifiziert, Bayes’schen Rahmen mit einer Effektgröße von 1,17 [95 %-KI 0,94–1,54]	In Übereinstimmung mit früheren Metaanalysen beobachteten wir einen kleinen, aber statistisch signifikanten Zusammenhang zwischen der regelmäßigen (im Vergleich zu nie) eingenommenem Pioglitazon und dem Blasenkrebsrisiko; die Kausalität ist jedoch nicht erwiesen und alternative Erklärungen können nicht ausgeschlossen werden

*KI* Konfidenzintervall

Die restlichen fünf Arbeiten beschäftigen sich alle mit dem Glitazon Pioglitazon. Nur eine Arbeit adressiert zusätzlich Rosiglitazon und schlussfolgert, dass die kumulative Anwendung von Pioglitazon oder Rosiglitazon nicht mit dem Auftreten von Blasenkrebs in dieser großen, gepoolten Multipopulationsanalyse assoziiert war. Hier handelt es sich um eine detaillierte Analyse von Verordnungs‑, Krebs- und Sterblichkeitsdaten von Menschen mit Typ-2-Diabetes aus 6 Populationen aus der gesamten Welt (British Columbia, Finnland, Manchester, Rotterdam, Schottland und dem UK Clinical Practice Research Datalink). Insgesamt gingen 1,01 Mio. Menschen mit 5,9 Mio. Personenjahren in die Statistik ein [13].

Die beiden aktuellsten Metaanalysen kommen hingegen zu dem Schluss, dass Pioglitazon das Risiko für das Auftreten von Blasenkrebs erhöht. Qu et al. berichtet, dass Pioglitazon das Risiko für Blasenkrebs in der europäischen Bevölkerung erhöht und ebenfalls bei Patienten die länger mit Pioglitazon (> 12 Monate) behandelt worden sind oder denen eine höhere kumulative Dosis (> 28.000 mg) verabreicht wurde, so dass diese möglicherweise besser bzgl. Blasenkrebs überwacht werden müssen [14]. Mehtälä et al. fanden einen kleinen, aber statistisch signifikanten, Zusammenhang zwischen der regelmäßigen Einnahme (im Vergleich zu nie) von Pioglitazon und dem Blasenkrebsrisiko. Allerdings räumen die Autoren ein, dass eine Kausalität bisher nicht gezeigt werden konnte und alternative Erklärungen somit nicht ausgeschlossen sind [2].

### Ergebnisse der translationalen Studien

Insgesamt konnten 8 translationale Studien inkludiert werden. Die Tab. 2 gibt einen Überblick über die Charakteristika dieser Arbeiten [7, 15–21].

Zwei experimentelle Untersuchungen adressierten explizit Rosiglitazon. Lubel et al. kam zu dem Ergebnis, dass Rosiglitazon über einen breiten Dosisbereich die Harnblasenkarzinogenese im OH-BBN-Modell von Ratten verstärkte [15]. Während Yang et al. schlussfolgerten, dass die PPAR-γ-Verstärkung in Blasenkrebs eine wichtige Rolle bei der Migration und Invasion von Blasenkrebszellen spielt sowie dass die Veränderung der PPAR-γ-Expression durch Rosiglitazon zu einer Veränderung der Migration und Invasion von Blasenkrebszellen führen könnte [16].

Zusammenfassend konnten fünf Studien keine kanzerogene Wirkung bzgl. Blasenkrebs von Glitazonen nachweisen. Zwei davon Studien postulierten sogar, dass Glitazonen einen möglichen therapeutischen Nutzen bei Blasenkrebs haben könnten [7, 17]. Hingegen kommen 3 Studien zu dem Schluss, dass Glitazone für die Kanzerogenese von Blasenkrebs eine entscheidende Rolle spielen. Sekeroglu et al. schlussfolgerten aus ihren Untersuchungen in der humanen Blasenkrebszelllinie hTU1, dass dies die erste Studie sei, die darauf hinweist, dass Pioglitazon DNA-Schäden und eine bösartige Transformation auslösen sowie die DNA-Reparaturkapazität in menschlichen Blasenzellen verringern bzw. verändern kann [21].

## Diskussion

Wir führten eine schnelle Evidenzanalyse bzgl. der Assoziation von Glitazonen und Blasenkrebs durch, auch um mögliche molekularbiologische Grundlagen darzustellen.

Die erste Frage, die sich dabei stellt, ist ob es diese Assoziation überhaupt gibt bzw. ist diese klinisch relevant? Seit dem Warnhinweis des BfArM sind 13 Jahre vergangen und es haben sich neue Daten ergeben, die in dieser Übersichtsarbeit dargestellt werden. Allerdings bleibt die Datenlage weiterhin uneindeutig.

### Methodische Probleme der inkludierten Studien

Die aktuellste Übersichtsarbeit kommt zu dem Schluss, dass es angesichts der vorliegenden Daten nicht sinnvoll ist, die Ergebnisse sehr heterogener Studien zusammenzufassen. Daher sollten konsequent durchgeführte Beobachtungsstudien konzipiert werden, um die Rolle der Anwendung von Pioglitazon für das Auftreten von Blasenkrebs korrekt einzuschätzen zu können [1]. Die wesentliche Problematik sind dabei die zahlreichen Störfaktoren, die bedacht werden müssen, um einen Zusammenhang korrekt zeigen zu können. Diese sind Nikotinabusus, Exposition von Umweltfaktoren, Lebensstil, kumulative Dosen sowie der zeitliche Aspekt der Einnahme. Anders ausgedrückt: Wie sieht die tatsächliche Exposition aus bzw. wie hoch ist diese? Bei diesem Faktor spielt zusätzlich die Compliance des Patienten eine entscheidende Rolle [1, 11]. Außerdem können Alter, Geschlecht, Ernährungsgewohnheiten, physische Aktivität, der Body Mass Index (BMI) und die Stoffwechsellage des Diabetes mellitus weitere Confounder darstellen [14]. Schlussendlich müssen in der Evaluation von Kanzerogenese noch die Familienanamnese bzw. die Genetik, die chemische Kanzerogenese inklusive Vortherapien, z. B. mit Cyclophosphamid und mikrobiologische Aspekte, z. B. Viren, berücksichtigt werden [12, 22]. Weiterhin muss in diesem Zusammenhang darauf hingewiesen werden, dass eine Assoziation und Kausalität unterschiedliche Dinge darstellen. Betrachtet man das Problem global bzw. die gesamte Population der Diabetes-mellitus-Typ-2-Erkrankten ist die Arbeit von Levin et al. hervorzuheben, da Daten von 1,01 Mio. Personen über 5,9 Mio. Personenjahren aus der gesamten Welt eingeschlossen worden sind. Es gab keine Hinweise auf einen Zusammenhang zwischen kumulativer Exposition gegenüber Pioglitazon und Blasenkrebs bei Männern (RR 100 Tage kumulativer Exposition 1,01; 95 %-KI 0,97–1,06) oder Frauen (RR 1,04; 95 %-KI 0,97–1,11) nach Anpassung auf Alter, Kalenderjahr, Dauer der Diabetes-mellitus-Erkrankung, Rauchen und jeglicher Verwendung von Pioglitazon, so dass insgesamt geschlussfolgert wurde, dass die kumulative Anwendung von Pioglitazon oder Rosiglitazon nicht mit dem Auftreten von Blasenkrebs in dieser großen, gepoolten Multipopulationsanalyse assoziiert war [13]. Betont werden muss, dass diese Publikation nach dem Warnhinweis des BfArM liegt. Zusammenfassend scheint die Problematik nicht bevölkerungsrelevant genauer für Patienten mit Typ-2-Diabetes mellitus zu sein. Aufgrund der Datenlage kann die Assoziation von Glitazonen mit Blasenkrebs maximal in einzelnen speziellen Patientengruppen relevant sein. Weiterhin sind Folgeuntersuchungen zum aktuellen Zeitpunkt aufgrund der Zulassungslage nur für Pioglitazon sinnvoll.

### Molekularbiologische Grundlagen der möglichen Assoziation

Die zweite Frage, die sich stellt, ist: Was sind die möglichen molekularbiologischen Grundlagen dieser Assoziation und gibt es einen möglichen Kausalitätsnachweis für die Kanzerogenese von Blasenkrebs durch Glitazon? Hier zeigt sich ein sehr heterogenes Bild bei den translationalen Studien und eine abschließende Schlussfolgerung kann nicht gezogen werden. Dabei sind die unterschiedlichen Untersuchungsmethoden bzw. Experimente hervorzuheben. Der erste Aspekt ist mit welchen Untersuchungsmaterialen die Experimente durchgeführt worden sind, z. B. im Tiermodell oder am humanen Präparat. Außerdem ist bei Studien mit Zelllinien entscheidend, welche Zelllinien verwendet worden sind, denn Blasenkrebs ist nicht gleich Blasenkrebs. Sekeroglu et al. betonen, dass sie die erste Studie liefern, die zeigt, dass Pioglitazon DNA-Schäden und eine bösartige Transformation auslösen sowie die DNA-Reparaturkapazität in menschlichen Blasenzellen verringern oder verändern kann [21]. Dabei verwenden die Autoren eine humane normale urotheliale Blasenzelllinie. Doch was passiert in diesen primären Urothellzellen oder wie stellt sich die DNA-Reparatur in bereits transformierten Zellen dar? Was passiert auf Transkriptom- oder Proteinebene? Insgesamt sind die Daten somit unzureichend für einen Kausalitätsnachweis. Der zweite wesentliche Aspekt sind Unterschiede der einzelnen PPAR-γ-Agonisten, so haben Pioglitazon und Rosiglitazon wesentlich andere pharmakokinetische Eigenschaften, die ebenfalls zu unterschiedlichen Assoziationen bzw. Wirkung auf Harnblasenzellen sowie Harnblasenkrebs beitragen könnten. Als Beispiel wird Pioglitazon in der Leber durch Hydroxylierung der aliphatischen Methylengruppen umfassend metabolisiert. Dies geschieht hauptsächlich über das Cytochrom P450 2C8, jedoch können andere Isoformen zusätzlich in geringerem Maße beteiligt sein. Drei der 6 identifizierten Metaboliten sind aktiv (M-II, M‑III und M‑IV). Wenn Aktivität, Konzentration und Proteinbindung berücksichtigt werden, tragen Pioglitazon und der Metabolit M‑III gleichermaßen zur Wirksamkeit bei. Auf dieser Basis entspricht der Beitrag von M‑IV zur Wirksamkeit in etwa dem 3‑Fachen der Wirksamkeit von Pioglitazon, wohingegen die relative Wirksamkeit von M‑II minimal ausgeprägt ist [23].

### Möglicher therapeutischer Nutzen von Glitazonen beim Blasenkrebs

Interessanterweise, postulieren zwei der inkludierten Studien, dass Glitazone sogar einen möglichen therapeutischen Nutzen bei Blasenkrebs haben könnten [7, 17]. Wang et al. zeigten so in ihren Ergebnissen, dass in den menschlichen Blasenkrebszelllinien T24 und 5637, dass der natürliche PPAR-γ-Ligand, 15d-PGJ2, die Zellproliferation reduziert und das Herunterregulieren von Survivin mit siRNA verstärkte signifikant die 15d-PGJ2-vermittelte Induktion der Zellapoptose [17]. Dies steht im Einklang mit den Ergebnissen, dass die Hochregulation von Nectin4 durch Rosiglitazon therapeutisch genutzt werden könnte [8]. Allerdings müssen dazu zahlreiche weitere Untersuchungen durchgeführt werden, die die oben genannten Probleme adressieren und stringent zwischen den einzelnen PPAR-γ-Agonisten unterscheiden. Weiterhin muss die Interaktion zwischen PPAR‑γ und Nectin4 sowie mit anderen Signalwegen sowie deren Relevanz für das Harnblasenkarzinom vollständig verstanden werden. Schlussendlich, sollten die präklinischen Daten sich weiter als vielversprechend darstellen, müssen erste klinische Untersuchungen detailliert geplant werden und die oben genannten Störfaktoren berücksichtigen sowie Nutzen-Risiko-Kalkulationen vornehmen, da insbesondere das kardiovaskuläre Risiko von Rosiglitazon nicht zu vernachlässigen ist. Einen ersten Anhaltspunkt für die Deskription der Interaktion der Signalwege liefert die Proteomanalyse von Shahid et al. Hier konnten in einer Charakterisierung des globalen Proteoms von humanen Blasenzellen 95 hochregulierte und 29 herunterregulierte Proteine (absolute log2-Fold-Change > 0,58 und *P*-Wert < 0,05) durch Pioglitazon identifiziert werden [20].

### Limitationen der eigenen Arbeit

Am Ende muss diskutiert werden, dass auch unsere Arbeit nicht ohne Limitationen ist, insbesondere da es sich um eine schnelle Evidenzsynthese handelt und nur eine Datenbank berücksichtigt worden ist. Außerdem hat es seit der letzten Suche eine wesentliche weitere Publikation gegeben, die hier noch genannt werden muss. Sie stellt eine Analyse der Food and Drug Administration Adverse Event Reporting System (FAERS) Datenbank da. Die Autoren fassten zusammen, dass Patienten, die mit Pioglitazon behandelt wurden, ein höheres Risiko für die Entwicklung von Blasenkrebs aufwiesen. Das männliche Geschlecht und das Alter führten zu mehr Blasenkrebsfällen unter Pioglitazon [24]. Diese Arbeit verdeutlicht noch einmal die essentielle Bedeutung der Berücksichtigung von Störfaktoren, um die Frage abschließend beantworten zu können.

### Schlussfolgerung

Zusammenfassend bleibt weiterhin unklar, ob Glitazone mit dem Auftreten bzw. Fortschreiten von Harnblasenkarzinomen assoziiert sind. In Folgestudien müssen zahlreiche Confounder adressiert werden, um diese Frage valide beantworten zu können. Wesentliches Ergebnis dieser Übersichtsarbeit ist, dass Glitazone hingegen einen therapeutischen Nutzen bei diesen Tumorpatienten haben könnten, insbesondere die Interaktion zwischen PPAR-γ-Aktivierung und Nectin4 scheint untersuchungswürdig zu sein.

**Tab. 2:** Überblick und Charakterisierung der eingeschlossenen translationalen Studien (*n* **=** 8)

Referenz	Studiendesign und Methodik	Hauptergebnisse	Schlussfolgerung der Autoren
Yoshimura et al., 2003 [7]	Untersuchung der Expression von PPAR‑α, -β, -γ, und in menschlichem Blasentumor und normalem Blasengewebe sowie die Auswirkungen von PPAR-Liganden; die Proben stammen von 170 Patienten mit Blasentumor und 20 mit normalem Blasengewebe; Expressionen wurden untersucht mittels RT-PCR und immunhistochemischer Methoden; Untersuchung der hemmenden Wirkung von PPAR-γ-Liganden auf die von Blasentumorgewebe abgeleitete Zelllinie	Das Ausmaß und die Intensität der immunreaktiven PPAR‑γ Polypeptide in Blasentumorzellen waren statistisch gesehen viel größer als die von normalen Zellen aus der Blase; es wurde eine Korrelation zwischen der PPAR-γ-Expression und dem Gewebetyp oder dem Fortschreiten des Blasenkrebses festgestellt; die PPAR-γ-Expression war in G3 von Blasenkrebs höher als in G1 und war höher fortgeschrittenem Krebs als im Frühstadium; PPAR-γ-Agonisten, Troglitazon, hemmt das Wachstum der Blasentumorzellen	PPAR‑γ wird in Blasentumoren exprimiert, und die Ergebnisse deuten darauf hin, dass PPAR-γ-Liganden eine starke antiproliferative Wirkungen gegen Blasentumorzellen haben. Daraus folgt, dass PPAR‑γ somit ein neues Ziel für die Behandlung von Blasentumoren sein könnte
Lubel et al., 2008 [15]	In einer ersten Studie, mit der festgestellt werden sollte, ob Rosiglitazon eine chemopräventive Wirkung hat, wurden weiblichen Fischer-344-Ratten 2‑mal wöchentliche Dosen von Hydroxybutyl(butyl)nitrosamin (OH-BBN), einem harnblasenspezifischen Karzinogen, über 8 Wochen verabreicht. Zwei Wochen nach der letzten OH-BBN-Dosis wurde den Ratten Rosiglitazon (50 mg/kgKG) täglich über die Magensonde für den Rest der Studie (7 Monate) zugeführt	Nur 57 % der mit OH-BBN behandelten Tiere entwickelten im Laufe der Studie tastbaren Harnblasenkrebs, während alle mit OH-BBN plus Rosiglitazon behandelten Ratten große Tumoren entwickelten (*p* < 0,01); Untersuchung auf PPAR‑γ durch Immunhistochemie zeigte in den Harnblasen der Ratten, dass das unbehandelte Blasenurothel und die präneoplastischen Läsionen eindeutig PPAP-γ-exprimierten; die niedrigste Dosis führte nicht zu einem signifikanten Anstieg der Tumorinzidenz (Rosiglitazon bei 0,4 mg/kg Körpergewicht [KG]/Tag, 64 %) oder des Tumorgewichts; Rosiglitazon allein (10 mg/kgKG/Tag), verabreicht in Abwesenheit von OH-BBN 10 Monate lang, führte nicht zur Bildung von Blasenkrebs	Rosiglitazon über einen breiten Dosisbereich verstärkte die Harnblasenkarzinogenese im OH-BBN-Modell bei Ratten
Yang et al., 2013 [16]	Die Genkopienzahl von PPAR‑γ in menschlichen Blasenkarzinomgewebeproben wurde durch Fluoreszenz-in-situ-Hybridisierung analysiert; die Migrations- und Invasionsfähigkeit von humanen Blasenkrebszelllinien mit unterschiedlichen PPAR-γ-Expressionsniveaus oder behandelt mit dem Thiazolidindion, Rosiglitazon, einem PPAR-γ-Agonisten und einem Antidiabetikum, wurde untersucht	Die PPAR-γ-Amplifikation war im Blasenkrebsgewebe im Vergleich zum normalen Urothel dramatisch erhöht (38,1 % versus 4,3 %; *p* = 0,0082); die humane Blasenkrebszelllinie 5637 mit starker PPARγ-Expression zeigte eine größere Fähigkeit zur Zellmigration und Invasion als die UMUC-3-Zelllinie mit schwacher PPAR-γ-Expression; das Ausschalten von PPAR‑γ in BCa-5637-Zellen führte zu einer verminderten Zellmigration, und die Aktivierung von PPAR‑γ mit Rosiglitazon förderte ihre Migration und invasive Fähigkeit	PPAR-γ-Verstärkung in Blasenkrebs könnte eine wichtige Rolle bei der Migration und Invasion von Blasenkrebszellen spielen; die Veränderung der PPAR-γ-Expression durch Rosiglitazon könnte zu einer Veränderung der Migration und Invasion von Blasenkrebszellen führen
Wang et al., 2014 [17]	Die kombinierte Wirkung von PPAR-γ-Agonisten und Survivin-Hemmung auf Blasenkrebszellen wurde untersucht; T24- und 5637-Zellen wurden 15d-PGJ2 behandelt, um festzustellen, ob 15d-PGJ2 eine hemmende Wirkung hat; die Lebensfähigkeit und Proliferation der Zellen wurden analysiert und Effizienz der Survivin-siRNA wurde mittels Western Blot-Analyse untersucht	Die Ergebnisse zeigten, dass in den menschlichen Blasenkrebszelllinien T24 und 5637, dass der natürliche PPAR-γ-Ligand, 15d-PGJ2, die Zellproliferation reduziert; Herunterregulieren von Survivin mit siRNA verstärkte signifikant die 15d-PGJ2-vermittelte Induktion der Zellapoptose	Ergebnisse legen nahe, dass die Kombination von 15d-PGJ2 und Inhibition von Survivin eine potenzielle Rolle bei der therapeutischen Behandlung von Blasenkrebs haben könnte
Yan et al., 2014 [18]	Untersuchung des Effekts von Troglitazone auf humane Blasenkrebszelllinie T24; benutzte Methoden: Zellkultur, Western Blot, siRNA-Knockdown, Immunfluoreszenz, Mikroskopie	Autophagieblockade führte zu einer Abschwächung der Troglitazone-abhängigen Apoptose; es wurde gezeigt, dass Troglitazone die Autophagie aktiviert	Troglitazone induzierte mehrere Arten von programmiertem Zelltod in Blasenkrebszellen
Yang et al., 2018 [19]	Normale Urothelzellen wurden von Sprague-Dawley-Ratten gewonnen; diese normalen Urothelzellen und J82-Blasenkrebszellen wurden mit unterschiedlichen Konzentrationen von Pioglitazon für verschiedene Zeiträume behandelt; die Zellproliferation wurde mit dem MTT-Assay getestet; Zellapoptose wurde mittels Durchflusszytometrie bewertet; Expressionen von p53, Cyclin D1, Bcl‑2 und Bax wurden durch qRT-PCR und Western Blots bestimmt	Pioglitazon hemmte die Proliferation und induzierte die Apoptose von normalen Urothelzellen, aber nicht von J82-Zellen, in einer zeit- und dosisabhängigen Weise; Behandlung mit Pioglitazon für bis zu 72 h führte nicht zu Veränderungen in der Expression von p53, Cyclin D1, Bcl‑2 und Bax in beiden untersuchten Zellen; Pioglitatone führte zu einer signifikanten Senkung des Proteingehalts von p53 und Cyclin D1 in J82-Zellen nach mehr als 192 h Behandlung	Pioglitazon förderte weder bösartige Veränderungen von normalen Urothelzellen noch stimulierte es die Proliferation von von J82-Zellen; Pioglitazon verringerte die Expression von p53 und Cyclin D1 in J82-Zellen nach Langzeitkultur, was darauf hindeutet, dass Pioglitazon für diabetische Patienten mit Blasenkrebs hilfreich sein könnte
Shahid et al. 2020 [20]	Markierte Flüssigchromatographie-Tandem-Massenspektrometrie-basierte Proteomikprofilierung Charakterisierung des globalen Proteoms von normalen menschlichen Blasenepithelzellen, die mit oder ohne Pioglitazon behandelt wurden	124 Proteine wurden identifiziert, die aufgrund der Pioglitazon-Behandlung differenziert exprimiert wurden; 95 hochregulierte und 29 herunterregulierte Proteine (absolute log2-Fold-Change > 0,58 und *P*-Wert < 0,05); Weitere zellbasierte Assays zeigten, dass Zelladhäsionsmoleküle, Marker des epithelial-mesenchymalen Übergangs und wichtige Signalwege durch die Behandlung mit Pioglitazon signifikant herunterreguliert wurden	Diese experimentellen Ergebnisse zeigten die proteomischen und biologischen Veränderungen, die in normalen Blasenzellen als Reaktion auf Pioglitazon auftreten; diese Ergebnisse lieferten ein Bild davon, wie das Blasenproteom durch Pioglitazon beeinflusst wird, was mögliche negative Auswirkungen von Diabetesmedikamenten und ihre Verbindungen zu Blasenfunktionsstörungen aufzeigt
Sekeroglu et al., 2021 [21]	Untersuchung der Auswirkungen von Pioglitazon auf die Zytotoxizität, DNA-Einzel- und Doppelstrangbrüche und Reparatur sowie neoplastische Transformation in menschlichen Blasenzellen (hTU1), die mit 10, 20 und 40 μM Pioglitazon für 24, 48 und 72 h behandelt worden; Methoden: Zellkultur, Zytoxizitäts‑, Transformationsassays, Immunfluoreszenz, Mikroskopie	Pioglitazon verringerte die Lebensfähigkeit der Zellen in einer konzentrationsabhängigen Weise; erhöhte Werte der Comet-Parameter zeigten, dass Pioglitazon und seine Metaboliten bei allen getesteten Konzentrationen signifikant DNA-Doppelstrangbrüche induzieren können; Pioglitazon kann auch eine bösartige Transformation menschlicher Blasenzellen auslösen	Dies ist die erste Studie, die darauf hinweist, dass Pioglitazon DNA-Schäden und eine bösartige Transformation auslösen sowie die DNA-Reparaturkapazität in menschlichen Blasenzellen verringern oder verändern kann; diese Ergebnisse legen nahe, dass Patienten mit Diabetes, die mit Pioglitazon behandelt werden, ein erhöhtes Risiko für Blasenkrebs haben

## Fazit für die Praxis

- Es bleibt weiterhin unklar, ob Glitazone in der Entstehung sowie Progression von Harnblasenkarzinomen eine Rolle spielen.
- In Deutschland ist, aufgrund der aktuellen Zulassungssituation, nur Pioglitazon relevant.
- Zahlreiche Störfaktoren, wie Alter, Geschlecht, Raucheranamnese oder chemische Exposition erschweren weitere Studien, müssen aber dringend berücksichtigt werden.
- Experimentell/translational gibt es Hinweise, dass Glitazone sogar einen therapeutischen Nutzen bei Harnblasenkrebspatienten haben könnten. Insbesondere die Interaktion zwischen PPAR-γ-Aktivierung und Nectin4 muss weiter untersucht werden.
